# Cluster analysis of articulatory trajectories in fluent nonword productions separates adults who stutter from fluent speakers

**DOI:** 10.1038/s41598-025-25829-0

**Published:** 2025-11-04

**Authors:** Andreas Leha, Nicole E. Neef, Susanne Dickhut, Daniela Ponssen, Annika Primassin, Alexandra Korzeczek-Opitz, Arun A. Joseph, Jens Frahm, Martin Sommer

**Affiliations:** 1 Department of Medical Statistics, University Medical Center, Göttingen, Germany; 2 Scientific Core Facility Medical Biometry and Statistical Bioinformatics, University Medical Center, Göttingen, Germany; 3 Department of Medical Bioinformatics, University Medical Center, Göttingen, Germany; 4 Department of Diagnostic and Interventional Neuroradiology, University Medical Center, Göttingen, Germany; 5 Department of Neurology, University Medical Center, Göttingen, Germany; 6 Biomedical NMR, Max-Planck-Institute for Multidisciplinary Sciences, Göttingen, Germany; 7 Present Address: Department of Health, FH Münster University of Applied Sciences, Muenster, Germany

**Keywords:** Real-time magnetic resonance imaging, Stuttering, Articulation, Trait and state markers; movement pattern analysis, Movement disorders, Neurodevelopmental disorders

## Abstract

Whether fluent-sounding utterances of adults who stutter are normally articulated is unclear. We asked 15 patients and 17 matched controls to utter a pseudoword while recording real-time MRI at 55 frames per second in a midsagittal plane, and we automatically clustered participants for distances between sites of articulation. Clustering was successful in 80% of the cases, indicating major differences in the movement patterns of fluent sounding utterances in both groups.

**Supplementary Information:**

The online version contains supplementary material available at 10.1038/s41598-025-25829-0.

At first glance, typical stuttering is an intermittent phenomenon. An archetypal example is a pupil speaking fluently to his peers in the schoolyard, but stuttering severely when asked to read a text aloud in the classroom. Indeed, this paroxysmal nature of stuttering generates misperceptions and increases the social burden of the disease^1^. Some therefore claim that the dysfluencies need to be taken care of, not the fluent parts of speech^2^. However, others postulate that stuttering is rarely silent, and that only a fraction of it is visible and audible on the surface, a view that compares it to an iceberg^3^. Hence, the border zone between fluent and dysfluent speech is disputed territory: it is unclear where fluent speech ends and where stuttering begins.

We wondered whether fluent-sounding speech of adults who stutter (AWS) is truly normal, a proposition that has been questioned in earlier reports^4^. We therefore used real-time MRI (rt-MRI)^5^ to characterize speech movements of the internal articulators in 15 AWS and 17 fluent speakers (FS). Participants uttered the nonword “natscheitideut” [natʃaitidɔyt] 15 times in a 3 T MRI scanner, while we recorded rtMRI videos at 55 frames per second in a midsagittal plane (Fig. 1a). Simultaneous audio recordings were made, and the corresponding oscillogram and spectrogram of the example utterance in Fig. 1a are shown in Fig. 1b and c. Only fluent productions were considered for analysis. We discarded all trials containing core symptoms of stuttering (repetitions of syllables and sounds, prolongations of sounds, and audible or silent blocking)^6^, and other disfluencies. For analysis, six profile lines were overlaid on each image using a MATLAB toolkit (Fig. 1d, Supplementary data Fig. 1): One to capture lip aperture, three in the anterior, one in the medial, and one in the posterior tongue regions. The movements of lip, tongue, and velum were examined at each profile line produced from the temporal intensity profiles (Fig. 1e). In detail, we measured lip aperture (LA), tongue tip retraction (TTR), tongue tip-to-teeth distance (TT-TD), tongue tip-to-alveolar ridge distance (TT-ARD), and tongue body-to-palate distances (TB-PD 1 and TB-PD 2).

**Fig. 1:**
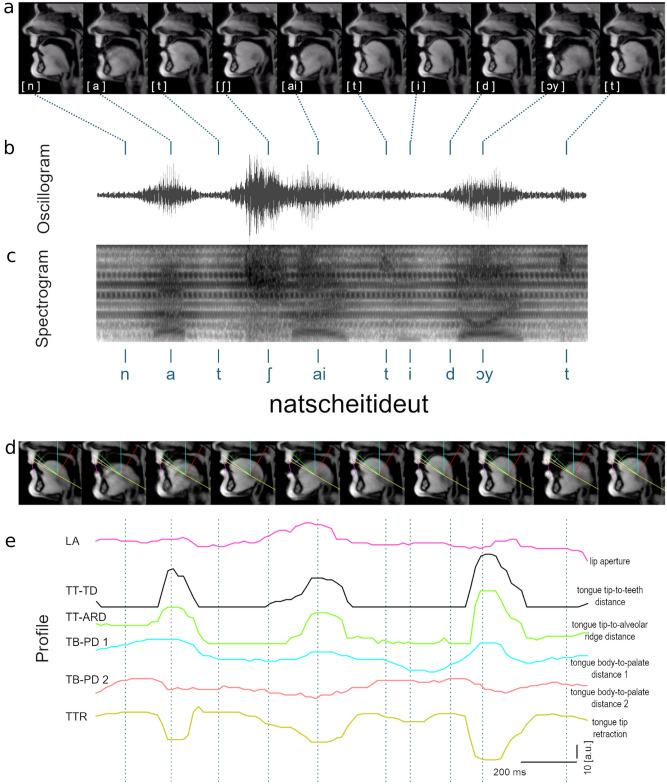
Kinematic trajectories extracted from real-time MRI. The top row displays midsagittal images of the vocal tract during producing the nonword “natscheitideut” (**a**). Below are the matching speech waveform (**b**), the sound spectrogram and segmentation lines of the corresponding phonemes (**c**). The second row of midsagittal images additionally shows the grid lines that served for extracting intensity profiles (**d**). Color-coded profile lines illustrate the kinematic trajectories of the articulators (**e**).

In a first step, we tested whether reproducible movement patterns of the articulators (lips, tongue and velum) could be recognized in fluent speech of AWS, and whether they differed from the fluent speech of FS. We used penalized flexible functional regression to model the movement of the tip of the tongue, lips and velum by group (AWS and FS) correcting for repeated measurements per subject. We assessed the group effect on the movement patterns. The patterns differed significantly between subjects (effective degrees of freedom, edf of s(participant) between 561 and 654, all p < 0.001) but only slightly so and showed a strong similarity across repetitions (Fig. 2a and b). The two groups only exhibited slightly different movement patterns in LA (edf of group 17, p < 0.001) and TB-PD 2 (edf of group 1, p = 0.016), but not in the remaining sites of articulation (Fig. 2c, Supplementary data Table 1). As a result, the movement patterns were distinguishable but still very similar across subjects, and only slight differences were observed between groups.

**Fig. 2:**
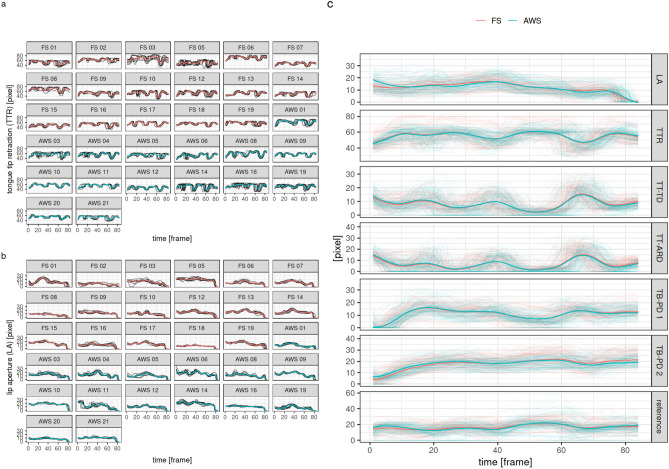
Modeled movement of articulators over time. The movement patterns were modeled using mixed-effect penalized functional regression models. (**a**,**b**) Individual model predictions for dimensions 1 and 2; thin lines = measured individual profiles for each repetition, thick lines = model predictions for each subject. (**c**) Group-level model predictions for all dimensions; thin lines = measured individual profiles, thick lines = group level model predictions. *AWS* adults who stutter, *FS* fluent speakers, *LA* lip aperture, *TTR* tongue tip retraction, *TT-TD* tongue tip-to-teeth distance, *TT-ARD* tongue tip-to-alveolar ridge distance, *TB-PD* tongue body-to-palate distance.

We went further and tested whether correlating movement patterns between articulators could be used to group the participants automatically. For this, we calculated all pairwise extents of spatiotemporal dissimilarity between the profile lines for each participant (Supplementary data Figs. 2 and 3). Automatic clustering of participants based on these spatiotemporal dissimilarities results in three clusters: (1) predominantly FS (n = 12), (2) predominantly FS (n = 5); (3) predominantly AWS (n = 14), Fig. 3a). A principal component analysis detected the same clusters (Fig. 3c). In this principal component analysis, all components beyond the second component explained less than 10% of the variation while the vast majority of the variation (75%) was explained by the first component (Fig. 3b).

**Fig. 3:**
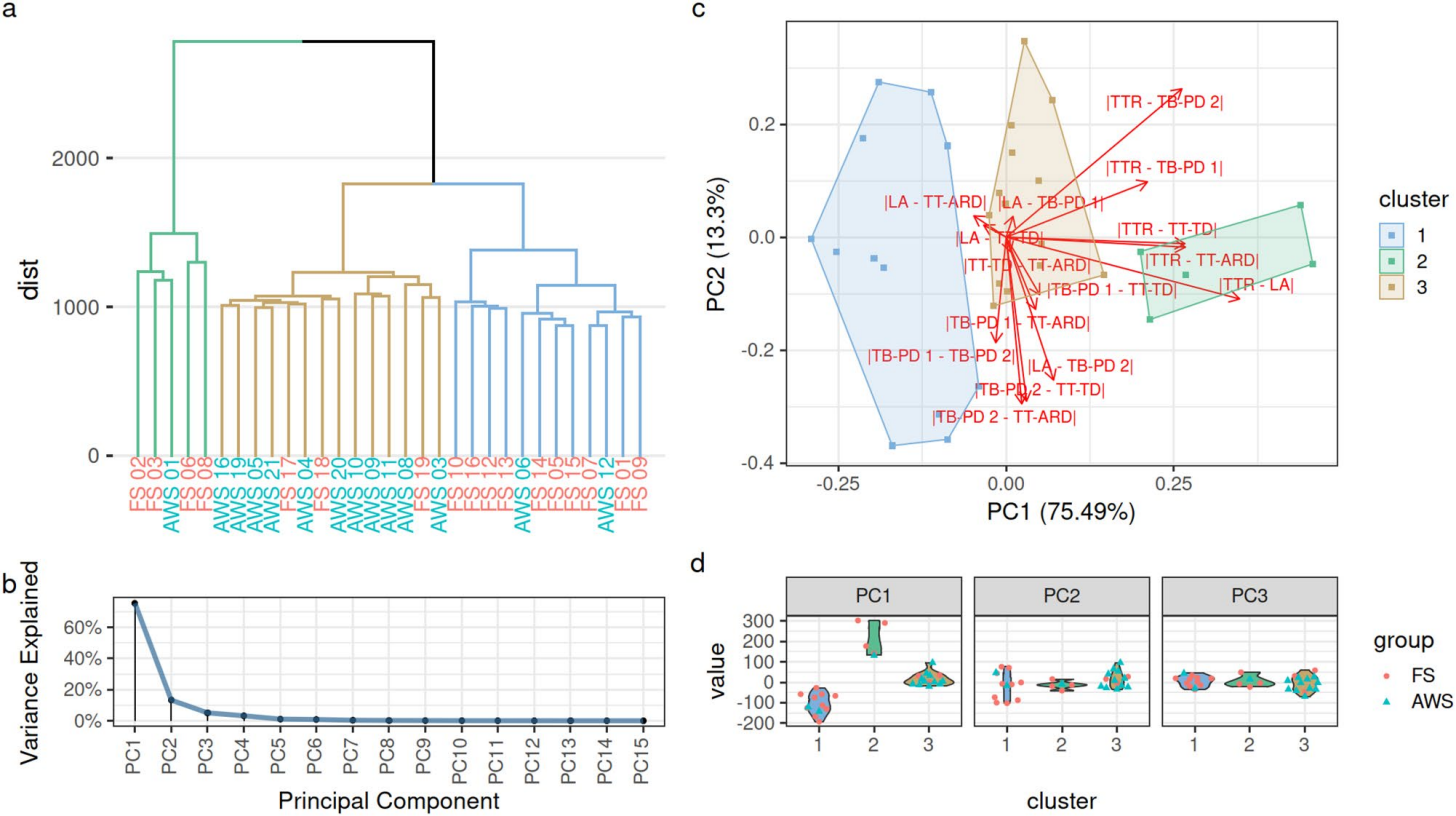
Fluent utterances differ between adults who stutter (AWS) and fluent speakers (FS). Based on the distances between movement patterns of different articulatory regions hierarchical clustering revealed three distinct clusters, of which one (brown) predominantly contained AWS while the other two (blue and green) predominantly contained FS (**a**). A principal component analysis (PCA) on these distances between movement patterns also shows a clear separation into the same three clusters (**c**). Most of the signal is captured in the first principal component (**b**). In this first principal component, the predominantly AWS cluster shows smaller values and separates the two predominantly FS clusters which show more extreme values (**d**). Overlaying the loadings on the PCA plot shows that the first principal component is mainly driven by distances between tongue-tip-retraction (TTR) and the other tongue regions (**c**). *LA* lip aperture; *TT-TD* tongue tip-to-teeth distance; *TT-ARD* tongue tip-to-alveolar ridge distance; *TB-PD* tongue body-to-palate distance.

Here, especially spatiotemporal dissimilarities between the TTR and the other articulators are loading onto the first principal component which is the component discriminating the clusters (Fig. 3d). It is not unexpected that TTR stands out from the other articulation sites as we quantified the extension of the tongue in that direction and not—as with the other articulation sites—the amount of air beyond the tongue (Supplementary data Fig. 1). However, since this analysis was done in an identical manner for both groups, we do believe that this does not introduce a bias when comparing AWS and FS.

We defined FS assigned to cluster 3 and AWS assigned to clusters 1 or 2 as ‘incorrectly allocated’, all others as ‘correctly allocated’. We compared epidemiological and questionnaire data between the correctly and incorrectly allocated adults, and we observed two significant differences: Correctly allocated participants had higher Beck Depression Inventory (BDI) score in comparison to the incorrectly allocated participants (5.1 ± 6.8 vs 0.50 ± 0.84; *p* = 0.04). In addition, we observed that the incorrectly clustered AWS had had stuttering therapy more recently than the correctly clustered AWS (years since last stuttering therapy 3.0 ± 1.7 vs 8.8 ± 6.7; *p* = 0.05). No further significant differences were observed between correctly and incorrectly clustered participants (Supplementary data Table 2).

To summarize, we used sophisticated kinematic analyses of real-time MRI image series capturing articulatory movements during fluent-sounding speech in AWS and FS. We observed reproducible movement patterns of the internal and external articulators which were not so much different between both groups. Spatiotemporal dissimilarities between movement patterns at different articulators, on the other hand, allowed clustering FS and AWS correctly in 80% of the cases. If we take a closer look at fluent-sounding utterances, group differences emerge. Hence, stuttering is not intermittent, but present most of the time.

To further investigate the contribution of the six different intensity profiles (LA, TT-TD, TT-ARD, TB-PD 1, TB-PD 2, TTR), we looked at the spatiotemporal dissimilarities between TTR and the other articulators which were crucial to the differentiation between AWS and FS (Supplementary data Fig. 4). Participants in cluster 3 (pre-dominantly AWS) exhibited dissimilarities that lay between the dissimilarities observed in the participants in cluster 1 and cluster 2 (pre-dominantly FS). Examples of the smallest and largest differences are shown in Supplementary data Fig. 2. Given the high relevance of TTR we tried to cluster on TTR alone, but this did not result in any clustering by group membership (AWS/FS) (Supplementary data Fig. 5).

## Discussion

We showed that the fine interplay between multiple articulators revealed characteristic movement dynamics of perceptually fluent speech in AWS. Spatiotemporal dissimilarities between distance measures of tongue, lips, and velum during articulatory movements distinguished AWS from FS. Previous studies identified various articulatory differences between AWS and FS, including differences for lip-jaw coordination variability, peak velocity to the following vowel, inter-articulator variability, and durations of lip- and jaw-closing movements^7–10^. These studies preferentially investigated the inter-articulatory coordination of jaw and lip movements because the internal articulators were previously notoriously difficult to investigate.

While the clustering identified a distinct group primarily composed of AWS, the intermediate position of this group in PCA space, situated between two fluent speaker clusters, and highlights the complementary nature of the two analyses and points to intermediate tongue kinematics in AWS. This may reflect compensatory strategies, reduced motor coordination, or temporal asynchrony in articulator gestures. The presence of two distinct clusters among fluent speakers further suggests that multiple articulatory strategies are at play. In the context of producing a novel and phonologically complex pseudoword in a loud environment, a task that inherently lacks familiar motor routines, these strategies may reflect different approaches to motor control under increased articulatory demands^11–13^. Hypoarticulation may reflect a relaxed, habitual motor pattern, while hyperarticulation may signal increased cognitive control aimed at producing accurate speech^15–17^. Most AWS appeared to fall between these extremes. One plausible interpretation is that restrictions in articulatory degrees of freedom, particularly in tongue movements, reflect an attempt to maintain control. It is tempting to speculate that the speech motor system of AWS is less adaptable to challenging speech conditions^19–21^. This interpretation underscores that even in perceptually fluent speech, measurable articulatory differences persist in AWS, likely reflecting deeper instabilities in speech motor control^22^.

An important factor contributing to these articulatory differences might be related to impaired kinaesthetic proprioception. De Nil and co-workers used an active movement task and succeeded in showing larger minimum displacements of the lips, tongue and jaw in AWS^23^. This evidence of reduced sensory acuity was only observed in the absence of visual feedback, and it was absent when studying minimal displacements of fingers. This is consistent with results from vibrotactile magnitude scaling in AWS^24^, and with reduced orofacial sensory abilities in children who stutter^25^.

Charting the border between fluent and dysfluent speech gives rise to vivid debates^26^. Our results support the iceberg hypothesis, which postulates that stuttering is always present, even if not perceptible on the surface. We conclude that the fluent-sounding speech of AWS is a topic worthwhile studying.

Depression is frequent among adolescents and young adults who stutter^27^, and may improve after speech therapy^28,29^. Interestingly, the few AWS who were misclassified into control clusters had lower depression scores and had received treatment more recently than those correctly classified in the AWS cluster. This pattern could indicate that reduced emotional burden or more recent therapeutic engagement may contribute to more “typical” or normalized articulatory patterns during fluent speech^30^. In other words, these individuals might exhibit articulatory dynamics more similar to fluent speakers, potentially reflecting treatment-related motor learning or reduced tension associated with lower affective distress. Overall, these findings suggest that the clustering structure not only captures group-level motor distinctions but may also be sensitive to individual differences in affective state and treatment history. This emphasizes the potential of data-driven kinematic analyses to identify subtle indicators of speech motor adaptation and recovery in AWS.

We conclude that a successful group allocation based on fluent-sounding utterances in adults who stutter suggests that the transition between fluent and stuttered speech is not abrupt, but rather a gradual continuum. However, studies in children and adolescents as well as pre- and post-treatment comparisons, will be essential to disentangle causative from adaptive phenomena.

## Online content

### Ethics statement

The protocol was approved by the University Medical Center Göttingen ethics committee, and we obtained written informed consent from each participant before any study-related procedure took place. All procedures were performed in accordance with the ethical standards of the 1964 Helsinki Declaration and its amendments, the most recent approved in 2024.

## Methods

### Participants

We investigated 15 subjects with persistent developmental stuttering (PDS). Clinical characteristics are shown in Table 1. They were recruited from the Göttingen and other stuttering peer support groups and by advertisement. Their data was compared with those from 17 matched FS with no personal or family history of stuttering. None of the participants had a history of any medical or neurological illness, and none were taking CNS-active drugs at the time of the study. As we only looked at fluent speech sections, all stuttered speech and other incorrect recordings were removed. Only fluent and error-free spoken pseudowords were analyzed, which is why the trials of some subjects had to be excluded completely, while only individual trials were excluded in other subjects. For example, two AWS were excluded because they did not produce enough fluent-sounding trials of the pseudoword, and a few others because they strictly and invariably used a modified, soft voice onset speech pattern learned in therapy shortly before participating in the study.

**Table 1:**
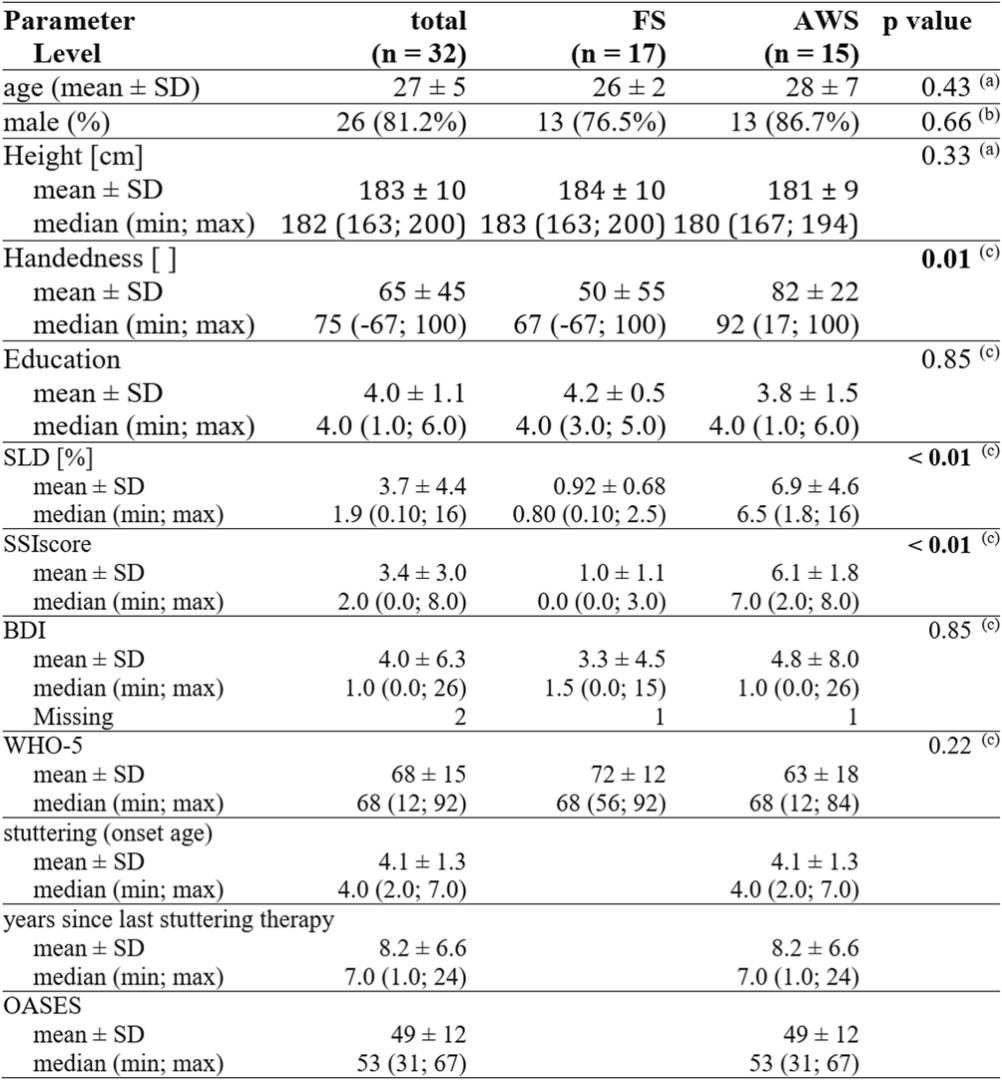
Epidemiological data of participants.

Handedness was assessed using an established questionnaire^31^, with scores reaching from full left-handedness (− 100) to full right-handedness (+ 100). Education was captured by the following scoring system (1, secondary/secondary school diploma; 2, high-school diploma/completed vocational training; 3, studies prior to bachelor’s degree/primary diploma; 4, completed bachelor’s degree/preliminary diploma; 5, diploma/Master’s degree; 6, Doctorate). The stuttering severity instrument (SSI) employs frequency of stutter-like-dysfluencies (SLD), i.e. percentage of syllables stuttered; duration, i.e. average length of the three longest stuttering events; physical concomitants, among these are distracting sounds, facial grimaces, head, arm and leg movements, and speech naturalness, to obtain a composite SSI total score. This ranges from 0 to 46 points, with higher scores indicating more stuttering. Stuttering age is the reported age of onset, height is the reported body height. The Overall Assessment of the Speaker’s Experience of Stuttering (OASES) is a 100-item questionnaire on the subjective impact of stuttering and a compound outcome score ranging from 0 to 5, with higher values indicating a greater impact^32^. The Beck Depression Inventory (BDI) is a questionnaire to measure the severity of depression with scores ranging from 0 to 63^33^. Higher total scores indicate more severe depressive symptoms. WHO-5 is a screening test for well-being with scores ranging from 0 to 5. Lower total scores indicate less well-being^34^. ^(a)^ Welch Two Sample t-test; ^(b)^ Fisher’s Exact Test for Count Data; ^(c)^ Brunner Munzel test.

We opted for a detailed analysis of the pseudoword "natscheitideut” mainly because of the articulation locations: It consists of the alveolar consonants [n], [d], and [t], and the postalveolar consonant [ʃ]. This is because the real-time MRI images and the sagittal cross-sectional image through the oral cavity are particularly good at depicting tongue movement. The articulation locations of the tongue tip corresponding to the alveolar and postalveolar consonants of this pseudoword, on the anterior maxillary incisor and on the hard palate behind the maxillary incisors, are thus visualized. The real-time MRI scan can therefore be used to analyze the relevant articulation positions of the pseudoword natscheitideut, embedded in its carrier phrase.

We obtained real-time MRI videos of 15 repetitions of the pseudoword. The first ten fluently spoken recordings per individual were extracted. Repetitions with stuttering or mis-pronounciations were not eligible for analysis. In addition, the first two recordings were regarded as training repetitions and also not analysed. Since maximally ten of the 15 repetitions were analysed, a sufficient number of completely fluently spoken runs could be evaluated even with relatively strongly stuttering subjects.

### Speech fluency assessment

The severity of the stuttering of the individual subjects was assessed by a qualified speech therapist using SSI-IV scores, which provide a reliable and valid norm-related grading^35^. The speech samples consisted of a conversation and a standard text, which was read aloud by the subjects. We used a linguistically balanced text of 422 words (650 syllables), “Der kleinste Schneesturm”, by Richard Brautigan. The conversation included motivating topics for the subject and covered 300 syllables^36^.

### Choice of linguistic material

Material obtained in an M.D. thesis^37^ comprised pseudowords involving different articulation loci, hence expanding the existing literature which focusses on bilabials visible to external movement tracking^8^. Four pseudowords (“gakscheitideuk”, “maptibibi”, “natscheitideut” and “mapscheitideup”) were visually presented on an MR-compatible screen, 15 times each in pseudorandomized order, embedding each word in a German carrier phrase ("Sa**g** XXX **b**itte").

Here we studied in detail the pseudoword “natscheitideut” [natʃaitidɔyt], which consists of the alveolar consonants [n], [d] and [t] and the postalveolar consonant [ʃ]. This focusses on tongue and velum movements well visible in real-time MRI videos in a midsagittal image orientation through the oral cavity. The articulation locations of the tip of the tongue on the anterior upper incisor and on the hard palate behind the upper incisors, which belong to the alveolar and postal convex consonants of this pseudoword, are thus represented. Such real-time MRI datasets allow one to analyse the relevant articulation positions of the pseudoword “natscheitideut”^38^.

Fluent speech sections were defined by the absence of typical stuttering characteristics such as repetitions of sounds, sound expansions or blockages. In addition, we discarded longer pauses or hesitations between two words or within a word if they led to a pause of more than 250 ms^39^. In addition, recordings were discarded if subjects made pronunciation errors (e.g. ‘natscheideut’ instead of ‘natscheitideut’).

### Real-time MRI

Technical details on real-time MRI are provided elsewhere^5^. In brief, participants were supine in a 3 T MRI system equipped with a 64-channel head coil (Siemens Healthcare, Erlangen, Germany). Participants wore ear plugs and MRI-compatible headphones connected to an MRI-compatible microphone (FOMRI III + , Optoacoustics Ltd, Israel). Real-time MRI was based on highly undersampled radial fast low-angle shot (FLASH) acquisitions. Recordings were in a midsagittal orientation covering the entire vocal tract, using an in-plane resolution of 1.4 × 1.4 mm^2^, a slice thickness of 8 mm, a field-of-view of 192 × 192 mm^2^ and a base resolution of 128 data samples per radial spoke. Acquisitions employed the following parameters: repetition time (TR) = 2.02 ms, echo time (TE) = 1.28 ms, flip angle = 5º, 9 spokes, measuring time per image = 18.18 ms corresponding to 55 frames per second.

### Film clip cutting

As explained in detail previously^40,41^ we extracted relevant sections from the individual real-time MRI videos using the programs Audacity (Audacity software copyright 1999–2019 Audacity Team) and VirtualDub (version 1.10.4, Avery Lee).

Using the Audacity program, it was possible to display the real-time MRI images as a spectrogram. The spectrogram shows three dimensions. Time is displayed on the x-axis, frequency on the y-axis, and the color intensity indicates the energy strength. The spectrogram made it possible to determine the pause in the plosives of the carrier phrase and thus the start and end points. A plosive is divided into the closure interval, in which, for example, with /g/ the back of the tongue moves to the hard palate and closes the space between them, and with /b/ the closure of the upper and lower lips. During the closure interval, the expiratory airflow is blocked behind the back of the tongue or behind the lips. This state is maintained for a short period of time. In the spectrogram, these voiced vowels display a so-called “voice bar” at below 500 Hz. The third phase, the closure release, is the sudden release with explosive noise. The closure burst lasts only 10–20 ms for voiced plosives. The plosive can be easily recognized and identified based on the closure interval, where only the “voice bar” is visible. (Pompino-Marschall 2009) and https://www.phonetik.uni-muenchen.de/studium/skripten/SGL/SGLKap2.html.

This makes it possible to precisely determine the starting points.

The program VirtualDub was chosen as a control. This program allows for visual and auditory viewing of the real-time MRI recordings. The time points determined with Audacity could be compared with the real-time MRI images. VirtualDub made it possible to view each image of the recording individually. Therefore, at the starting point, the dorsum of the tongue had to be on the soft palate, and at the end point, the lip closure for the [b] sound had to have just been completed. In this way, possible errors in the determination using the spectrogram could be improved upon.

We displayed the real-time MRI recordings as a three-dimensional spectrogram (Fig. 1c): time is displayed on the X axis, frequency on the Y axis, and colour intensity indicating the energy level on the Z axis. In the spectrogram, the plosives and thus the start and end points ([g] and [b], see above) could be detected. A plosive is subdivided into the first phase of closure formation in which, for example, the / g / of the back of the tongue migrates to the hard palate and closes the intra-oral space, or in the case of / b / the closure of the upper and lower lip. In the second, closing phase, the expiratory air flow is stowed behind the back of the tongue or behind the lips. Over a short period of time, this condition is maintained. In the spectrogram you can see in these voiced vowels a so-called “voice bar” at below 500 Hz. The third phase is the sudden solution under explosive noise. The shutter sound takes only 10–20 ms for voiced plosives. The plosive is easy to recognize and to determine based on the closing phase, which can be seen on the “voice bar” (https://www.phonetik.uni-muenchen.de/studium/skripten/SGL/SGLKap2.html).

Hence, it is possible to exactly determine the starting point. We used the open source program VirtualDub as internal control. With this program one can visually and audibly review the real-time MRI image series. The times determined with Audacity could be compared with the images of the real-time MRI. With VirtualDub one can view each image individually. At the starting point from the carrier phrase, therefore, the back of the tongue had to be on the soft palate, and at the end point, the lip closure for the [b] had just been completed. Thus, possible errors of the determination with the spectrogram could be corrected.

### Processing of film clips

We further processed the recordings in the MATLAB R2017b program with Image Processing and Signal Processing Toolbox (The Mathworks, Inc., Natick, Massachusetts, USA), which was specially developed for the dynamic data analysis of real-time MRI videos^42^. The toolbox places a grid over an image of the real-time MRI video. All pixels on which the grid is located can now be viewed as a function of time. The grid consists of a baseline, which is drawn between two fixed points in the image. Here we used the upper frontal edge of the cervical vertebrae 4. A second fixed point was the transition from the anterior incisor to the hard palate, and the baseline was drawn between these two fixed points. Five lines of half the baseline length were drawn from the midpoint of the baseline at angles of 7.5°, 15°, 30°, 60°, 90°, 120° and 150° counted clockwise. The baseline itself counted as 0° and 180°, respectively. Now the pixel values of the individual lines of the grid were analysed over the duration of the real-time MRI scan, and a line profile was created showing the pixel resolution of a single grid line on the Y axis and the time change on the X axis. This way, each line profile shows the pixels of each gridline over time. These line profiles can be used for further evaluation^42^.

Indeed, this type of analysis was successful in visualizing the positions of the articulators tongue, lips, and velum when consonants are spoken^38^. In addition to the alveolar and postalveolar consonants of the pseudoword, the carrier phrase contained the velar consonant [g] and the bilabial consonant [b] of the two auxiliary words “sag” (“say”) and “bitte” (“please”). Therefore, we modified the grid:

Starting from the baseline, three lines were drawn to obtain information about tongue tip movement for the alveolar and postalveolar consonants. Starting from the midpoint of the baseline, these three lines were 0 degrees (i,e, anterior part of the baseline), 7.5 degrees, and 15 degrees apart. In Fig. 1d and Supplementary data Fig. 1 these lines are yellow, white and green. Information about the velar consonant [g] with the place of articulation of the back of the tongue, which abuts the hard palate, should be found by a line at a distance of 60 degrees, clockwise from baseline. This line is blue in the Fig. 1. Another line 90 degrees clockwise from baseline was placed to assess velum movement. It is shown in red in Fig. 1d. One more line is placed between the center of the subject’s upper lip and the center of the lower lip, shown in purple in Fig. 1d. In this way, the lip movement at the bilabial consonant [b] can be detected. Finally, the last line is the posterior part of the baseline starting from the center point to the spine. It does not record any relevant articulation and was not used in the analyses presented here, but was indicative of the extent of head movement during the recording. This line is the posterior half of the yellow line in the figure.

For the lines at 7.5° and at 15°, we have seen that the “thin white line” connecting the palate with the upper incisor does not move throughout the entire word. Thererefore, we could measure the space between the tip of the tongue and the “palate and incisor” (Supplementary data Fig. 1).

However, the anterior baseline (yellow) does not intersect the palate at a stable anatomical reference point. The next potential interface, the upper lip, is itself mobile, making it unsuitable as a fixed reference. Since our aim was in addition to isolate tongue movement independently of other articulatory structures, we decided, after extended discussions, to include the lower conture, the tip of the tongue, in our analysis. Accordingly, TTR was defined as the distance between the tongue tip and the midpoint of the tongue at 0° orientation (Supplementary data Fig. 1).

During the recording some subjects moved their head between speaking the consecutive pseudowords. For this reason, the grid was readjusted for each subject at every new repetition. The starting point for the grid adaptation was the first frame of the section to be analysed with the articulation position of the consonant "g".

### Processing of line profiles

The measurement of gap widths or distances from reference points in the line profiles was accomplished semi-automatically using in-house developed software (Supplementary data Fig. 6). Matter was differentiated from air based on the grey value of the pixels. Constrained three-component Gaussian mixture models were fit to the distribution of the grey values to classify the areas into air (dark), matter (light) and uncertain (grey) parts. The black component of the mixture distribution was used to set a threshold for image binarization. Detected gaps were registered over time by overlap to previous gap pixels. The grey-value threshold and the choice of gap to retain in case of multiple detected gaps could be manually overridden in an interactive application. Thus, the gaps can be accurately detected because the pixel values of the tongue, lip and hard palate clearly differ from the pixel value of the air space between the anatomical structures (Supplementary data Fig. 7).

### Statistical analysis

As stated previously^40,41^, epidemiological and questionnaire results were compared between groups using unpaired, two-tailed t-test, Brunner-Munzel test, or Fisher’s exact test, as appropriate.

From all considered samples the median time needed for the uttering of the pseudoword was calculated. All line profiles were then stretched or compressed to that median time. Time is given in frames in this data, as the movement was recorded at 55 frames per second, so the median time corresponds to a median number of frames. All line profiles were then again read off at the same number (the median) of frames (time points) using linear interpolation.

To compare movement patterns between AWS and FS, the line profiles were scaled to the median duration. To ensure comparability in the time resolution, the line profiles were sampled at an equal number of time points using linear interpolation. Penalized flexible functional regression was applied to compare movement patterns between AWS and FS, the line profiles were scaled to the same duration and penalized flexible functional regression was applied to assess group effects in the time curves while accounting for repeated measurements of each subject by adding “subject” as random time-varying effect. The factor “group” was added as ordinal factor so that the significance test can be interpreted as testing for difference in the mean course. Modelled curves per subject as well as per group were visualized.

For the cluster analysis the pairwise extents of spatiotemporal dissimilarity between the sites of articulation LA: lip aperture; TTR: tongue tip retraction; TT-TD: tongue tip-to-teeth distance; TT-ARD: tongue tip-to-alveolar ridge distance; TB-PD: and tongue body-to-palate distance were calculated as the L2-metric approximated by Simpson’s rule per subject and repetition (Supplementary data Fig. 2). The resulting extents of spatiotemporal dissimilarity inform about the spatiotemporal dissimilarity of sites, and were then averaged per subject (Supplementary data Fig. 3). Energy distances^43^ between patients were then calculated and used as basis for hierarchical clustering using Ward’s minimum variance method and complete linkage. The cindex^44^ was used to identify an optimal number of clusters. Fisher’s exact test was used to test the independence between the clustering and the grouping (FS and AWS). In addition to the hierarchical clustering, a principal component analysis (PCA) was conducted on the extents of spatiotemporal dissimilarity between the sites of articulation. The loadings of the distances on the components have been visualized.

Having observed the importance of the TTR in the clustering we also attempted clustering on TTR alone. Here instead of using the spatiotemporal dissimilarity between two sites of articulation we used the integral of the TTR line profile (in our wording the spatiotemporal dissimilarity between TTR and a hypothetical gap staying closed throughout the utterance) as basis for the clustering.

All analyses of the extracted images were performed in R (version 3.6.1; R Core Team 2018) using the packages EBImage (version 4.25.0)^45^ for image manipulation, shiny (version 1.3.2)^46^ to support the semi-automated gap detection, tidyfun (version 0.0.82)^47^ and refund (version 0.1.21)^48^ for the handling and modelling of the functional data, Rfast (version 1.9.9) for the energy distances between matrices, NbClust (3.0.1) for the determination of an optimal number of clusters.

## Supplementary Information

Supplementary Information 1.

Supplementary Information 2.

Supplementary Information 3.

Supplementary Information 4.

Supplementary Information 5.

Supplementary Information 6.

Supplementary Information 7.

Supplementary Information 8.

Supplementary Information 9.

Supplementary Information 10.

## Data Availability

Pseudonymized data supporting the findings of this study are available from the corresponding author upon reasonable request and within the limits of the informed consent, as the video and audio are considered identifying information.
