# Supplementary material for: Cluster analysis of articulatory trajectories in fluent nonword productions separates adults who stutter from fluent speakers
Source: Sci Rep. 2025 Nov 4;15:38465. doi: 10.1038/s41598-025-25829-0 (PMC12586618; doi:10.1038/s41598-025-25829-0)
Supplement: Supplementary file 1 — Supplementary Information 1. [file 41598_2025_25829_MOESM1_ESM.pdf]

Legends to supplementary material

SupplementaryTab. 1: Model coefficients from penalized flexible functional regression models per place of articulation as visualized in Fig 2. The participant is added as random time-varying effect. The test for group effects can be interpreted as testing for difference in the mean time courses. edf: estimated degrees of freedom, Ref.df: reference degrees of freedom.

SupplementaryTab. 2: Epidemiological data of correctly and incorrectly clustered participants. See legend of Table 1 for details.

SupplementaryFig. 1: Seventh image from the second row of midsagittal images from Fig. 1 D, magnified for easier reading. It shows the grid lines that served for extracting intensity profiles. Color-coded profile lines illustrate the kinematic trajectories of the articulators. Solid parts are the quantified segments.

SupplementaryFig. 2: Articulation patterns for representatives of each cluster. Median articulation patterns for one representative participant per cluster are shown. Time is on the X axis, the measured articulation pattern on the Y axis. Each panel shows three (one per representative) articulation patterns for two sites of articulation (red always TTR). The shaded area corresponds to the measured distance. AWS: adults who stutter; FS fluent speakers; LA: lip aperture; TTR: tongue tip retraction; TT-TD: tongue tip-to-teeth distance; TT-ARD: tongue tip-to-alveolar ridge distance; TB-PD: tongue body-to-palate distance.

SupplementaryFig. 3: Pairwise extents of spatiotemporal dissimilarity in movement patterns between the sites of articulation studied here. Each panel shows data from one individual. The distances are measured as the L2-metric approximated by Simpson's rule. Distances of repetitions were averaged. Darker colours for lower distances. AWS: adults who stutter; FS fluent speakers.

SupplementaryFig. 4: Pairwise distances in movement patterns between TTR and the other studied sites of articulation. Each dot shows the distance for one participant. The distances are measured as the L2-metric approximated by Simpson's rule. Distances of repetitions were averaged. Red dots: FS; blue dots: AWS**.** AWS: adults who stutter; FS fluent speakers; LA: lip aperture; TTR: tongue tip retraction; TT-TD: tongue tip-to-teeth distance; TT-ARD: tongue tip-to-alveolar ridge distance; TB-PD: tongue body-to-palate distance

SupplementaryFig. 5: Tongue tip retraction (TTR) alone does not discriminate AWS and FS. Clusters obtained by hierarchical clustering on the average area under the TTR articulation pattern alone do not discriminate between the groups (AWS and FS). AWS: adults who stutter; FS fluent speakers.

SupplementaryFig. 6: In-house software to refine gap detection. The central image displays (from bottom to top) the extraction of the MRI video, a pixel map of the detected air, an overlay of the pixel map over the extracted MRI image, the measured gap width (in pixels). The left side bar shows the data selector, the right side bar shows the parameters that can be adjusted.

SupplementaryFig. 7: Examples of semi-automated gap detection and gap measurement in each of the extracted sites of articulation. In each image, the bottom panel shows the extracted line profile, the third panel show a pixel map of the detected air, the second panel shows an overlay of the quantified parts over the extracted line profile, and the top panel shows the measured gap width/lengths (in pixels) which are the line profiles used in the downstream analyses.
