# Supplementary material for: Cluster analysis of articulatory trajectories in fluent nonword productions separates adults who stutter from fluent speakers
Source: Sci Rep. 2025 Nov 4;15:38465. doi: 10.1038/s41598-025-25829-0 (PMC12586618; doi:10.1038/s41598-025-25829-0)
Supplement: Supplementary file 2 — Supplementary Information 2. [file 41598_2025_25829_MOESM2_ESM.pdf]

	Constant intercept:			Smooth terms & functional coefficients:			
	**Estimate**	**Std. Error**	**P value**	**Term**	**edf**	**Ref.df**	**P value**
**TTR**	55.0	0.44	< 0.001	Intercept(yindex)	16.0	19.0	< 0.001
				group(yindex)	1.0	1.0	0.460
				s(participant)	654.0	798.0	< 0.001
**LA**	12.0	0.30	< 0.001	Intercept(yindex)	17.0	19.0	< 0.001
				group(yindex)	17.0	19.0	**< 0.001**
				s(participant)	561.0	798.0	< 0.001
**TB-PD 1**	11.0	0.28	< 0.001	Intercept(yindex)	17.4	19.0	< 0.001
				group(yindex)	1.5	1.6	0.675
				s(participant)	579.1	798.0	< 0.001
**TB-PD 2**	18.0	0.32	< 0.001	Intercept(yindex)	17.0	19.0	< 0.001
				group(yindex)	1.0	1.0	**0.016**
				s(participant)	525.0	798.0	< 0.001
**TT-TD**	8.20	0.23	< 0.001	Intercept(yindex)	17.2	19.0	< 0.001
				group(yindex)	1.1	1.1	0.186
				s(participant)	624.4	773.0	< 0.001
**TT-ARD**	6.50	0.21	< 0.001	Intercept(yindex)	17.0	19.0	< 0.001
				group(yindex)	1.0	1.0	0.096
				s(participant)	646.0	773.0	< 0.001

SupplementaryTab. 1: Model coefficients from penalized flexible functional regression models per place of articulation as visualized in Fig 2. The participant is added as random time-varying effect. The test for group effects can be interpreted as testing for difference in the mean time courses. edf: estimated degrees of freedom, Ref.df: reference degrees of freedom.
