# Supplementary material for: Cluster analysis of articulatory trajectories in fluent nonword productions separates adults who stutter from fluent speakers
Source: Sci Rep. 2025 Nov 4;15:38465. doi: 10.1038/s41598-025-25829-0 (PMC12586618; doi:10.1038/s41598-025-25829-0)
Supplement: Supplementary file 3 — Supplementary Information 3. [file 41598_2025_25829_MOESM3_ESM.pdf]

| **Parameter**<br>**Level** | **incorrectly allocated (n = 6)** | **correctly allocated (n = 25)** | **p value** | **adjusted p value** |
|---|---|---|---|---|
| age (mean ± sd) | 26 ± 3 | 27 ± 6 | 0.54 | 1.00 (a) |
| height (mean ± sd) | 182 ± 8 | 183 ± 10 | 0.86 | 1.00 (a) |
| male (%) | 5 (83.3%) | 20 (80.0%) | 1.00 | 1.00 (b) |
| Handedness | | | 0.89 | 1.00 (c) |
| mean ± sd | 56 ± 61 | 66 ± 42 | | |
| median (min; max) | 71 (-66; 100) | 75 (-67; 100) | | |
| Education | | | 0.66 | 1.00 (c) |
| mean ± sd | 4.2 ± 0.8 | 3.9 ± 1.2 | | |
| median (min; max) | 4.0 (3.0; 5.0) | 4.0 (1.0; 6.0) | | |
| SLD [%] | | | 0.87 | 1.00 (c) |
| mean ± sd | 2.4 ± 2.3 | 4.0 ± 4.8 | | |
| median (min; max) | 1.7 (0.10; 6.8) | 1.8 (0.10; 16) | | |
| SSIscore | | | 0.86 | 1.00 (c) |
| mean ± sd | 14 ± 6 | 15 ± 11 | | |
| median (min; max) | 15 (4.0; 20) | 13 (0.0; 37) | | |
| Missing | 1 | 0 | | |
| BDI | | | **0.04** | 0.56 (c) |
| mean ± sd | 0.50 ± 0.84 | 5.1 ± 6.8 | | |
| median (min; max) | 0.0 (0.0; 2.0) | 3.0 (0.0; 26) | | |
| Missing | 0 | 2 | | |
| WHO-5 | | | 0.15 | 1.00 (c) |
| mean ± sd | 75 ± 10 | 66 ± 16 | | |
| median (min; max) | 72 (64; 92) | 68 (12; 92) | | |
| stuttering (age)* | | | 0.44 | 1.00 (c) |
| mean ± sd | 3.3 ± 0.6 | 4.2 ± 1.5 | | |
| median (min; max) | 3.0 (3.0; 4.0) | 4.0 (2.0; 7.0) | | |
| years since last stuttering therapy* | | | **0.05** | 0.62 (c) |
| mean ± sd | 3.0 ± 1.7 | 8.8 ± 6.7 | | |
| median (min; max) | 4.0 (1.0; 4.0) | 8.0 (1.0; 24) | | |
| OASES* | | | 0.88 | 1.00 (c) |
| mean ± sd | 52 ± 9 | 50 ± 13 | | |
| median (min; max) | 53 (43; 61) | 53 (32; 67) | | |

**ExtendedTab. 2: Epidemiological data of correctly and incorrectly clustered participants.**

See legend of Table 1 for details.
