# Supplementary material for: Cluster analysis of articulatory trajectories in fluent nonword productions separates adults who stutter from fluent speakers
Source: Sci Rep. 2025 Nov 4;15:38465. doi: 10.1038/s41598-025-25829-0 (PMC12586618; doi:10.1038/s41598-025-25829-0)
Supplement: Supplementary file 4 — Supplementary Information 4. [file 41598_2025_25829_MOESM4_ESM.pptx]

## Slide 1
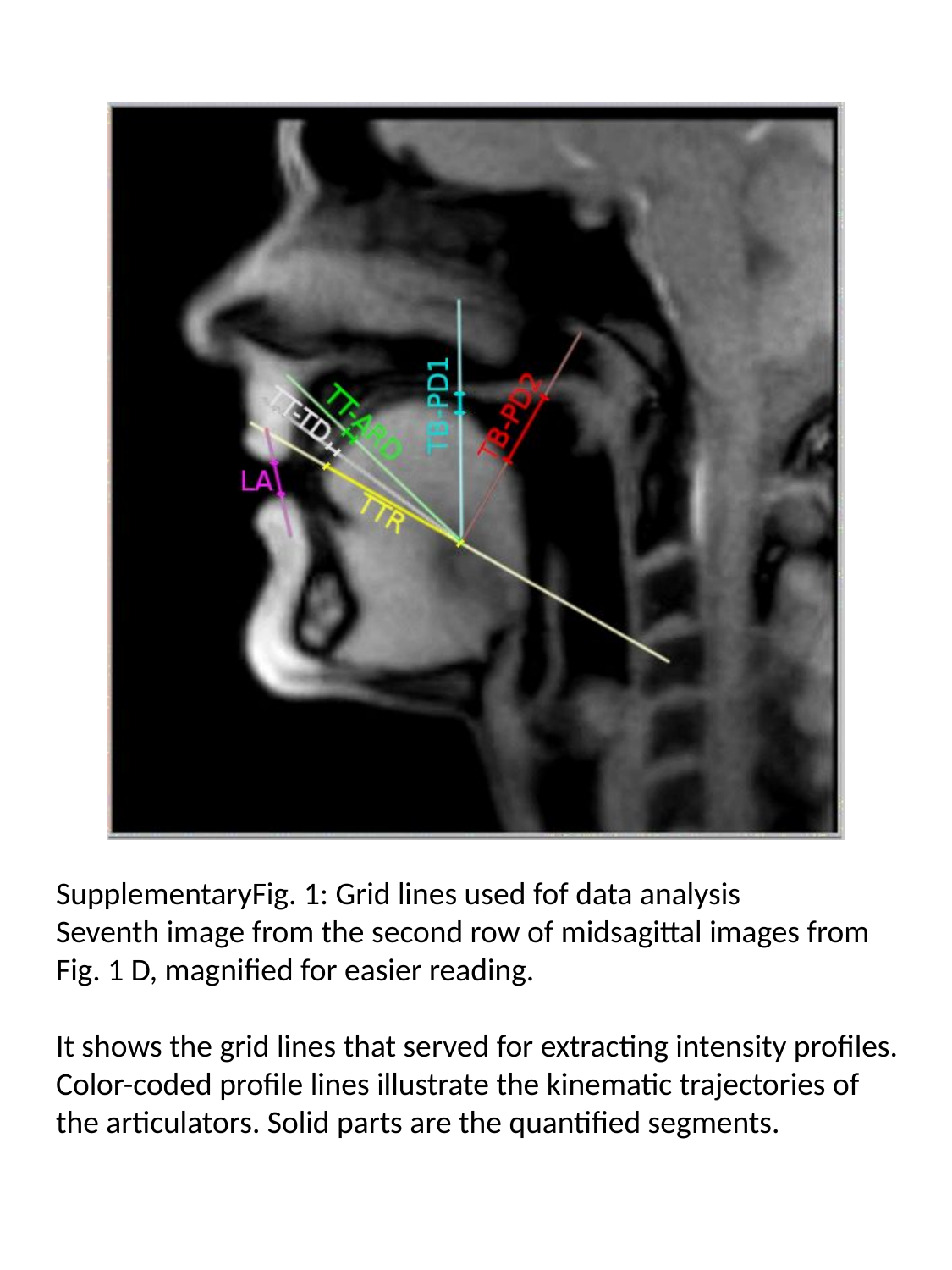

SupplementaryFig. 1: Grid lines used fof data analysis
Seventh image from the second row of midsagittal images from
Fig. 1 D, magnified for easier reading.
It shows the grid lines that served for extracting intensity profiles.
Color-coded profile lines illustrate the kinematic trajectories of
the articulators. Solid parts are the quantified segments.
