# Supplementary material for: Cluster analysis of articulatory trajectories in fluent nonword productions separates adults who stutter from fluent speakers
Source: Sci Rep. 2025 Nov 4;15:38465. doi: 10.1038/s41598-025-25829-0 (PMC12586618; doi:10.1038/s41598-025-25829-0)
Supplement: Supplementary file 5 — Supplementary Information 5. [file 41598_2025_25829_MOESM5_ESM.pptx]

## Slide 1
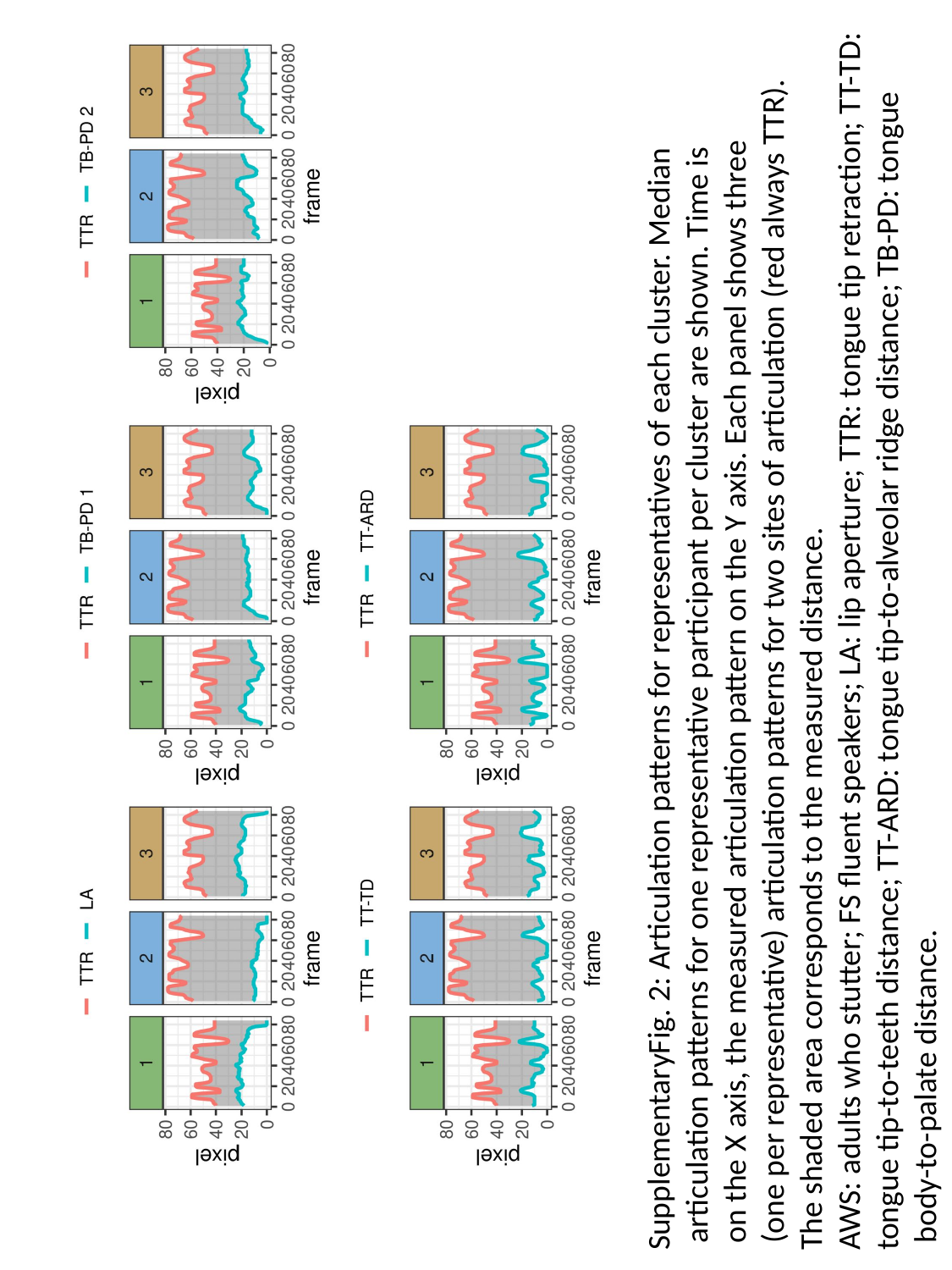

SupplementaryFig. 2: Articulation patterns for representatives of each cluster. Median
 articulation patterns for one representative participant per cluster are shown. Time is
 on the X axis, the measured articulation pattern on the Y axis. Each panel shows three
 (one per representative) articulation patterns for two sites of articulation (red always TTR).
The shaded area corresponds to the measured distance.
AWS: adults who stutter; FS fluent speakers; LA: lip aperture; TTR: tongue tip retraction; TT-TD:
tongue tip-to-teeth distance; TT-ARD: tongue tip-to-alveolar ridge distance; TB-PD: tongue
 body-to-palate distance.
