# Supplementary material for: Cluster analysis of articulatory trajectories in fluent nonword productions separates adults who stutter from fluent speakers
Source: Sci Rep. 2025 Nov 4;15:38465. doi: 10.1038/s41598-025-25829-0 (PMC12586618; doi:10.1038/s41598-025-25829-0)
Supplement: Supplementary file 6 — Supplementary Information 6. [file 41598_2025_25829_MOESM6_ESM.pptx]

## Slide 1
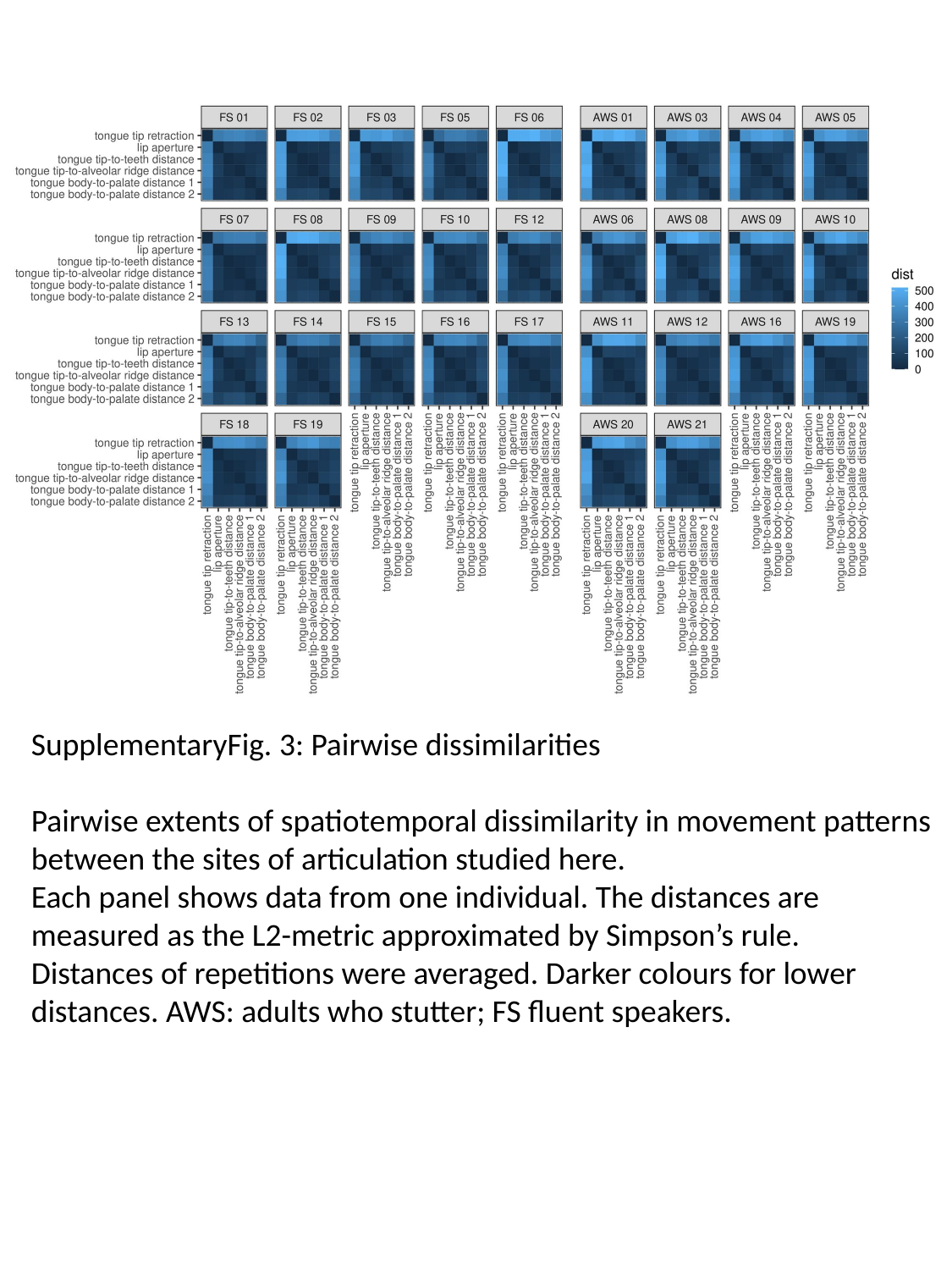

SupplementaryFig. 3: Pairwise dissimilarities
Pairwise extents of spatiotemporal dissimilarity in movement patterns
between the sites of articulation studied here.
Each panel shows data from one individual. The distances are
measured as the L2-metric approximated by Simpson’s rule.
Distances of repetitions were averaged. Darker colours for lower
distances. AWS: adults who stutter; FS fluent speakers.
