# Supplementary material for: Cluster analysis of articulatory trajectories in fluent nonword productions separates adults who stutter from fluent speakers
Source: Sci Rep. 2025 Nov 4;15:38465. doi: 10.1038/s41598-025-25829-0 (PMC12586618; doi:10.1038/s41598-025-25829-0)
Supplement: Supplementary file 7 — Supplementary Information 7. [file 41598_2025_25829_MOESM7_ESM.pptx]

## Slide 1
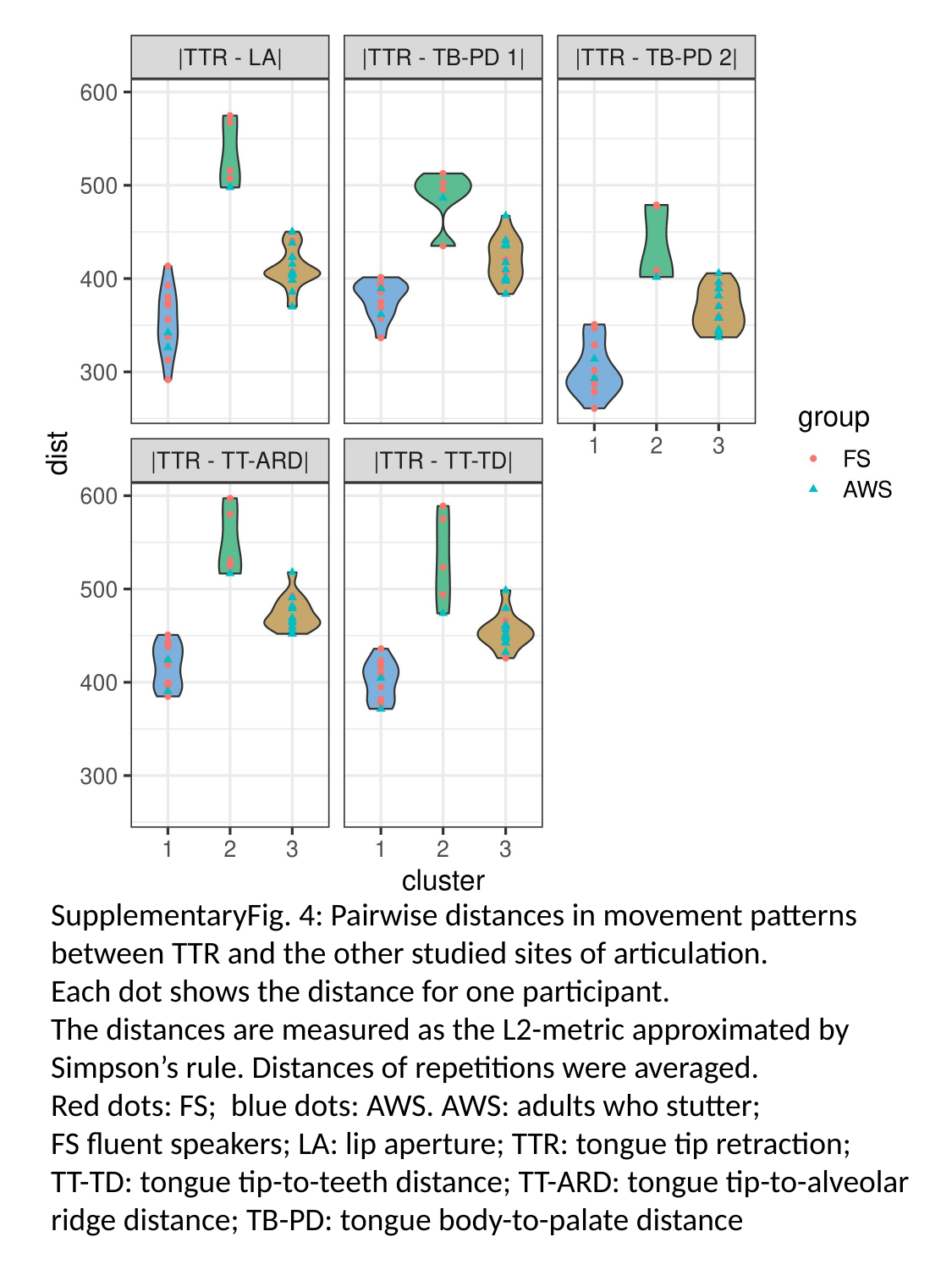

SupplementaryFig. 4: Pairwise distances in movement patterns
between TTR and the other studied sites of articulation.
Each dot shows the distance for one participant.
The distances are measured as the L2-metric approximated by
Simpson’s rule. Distances of repetitions were averaged.
Red dots: FS; blue dots: AWS. AWS: adults who stutter;
FS fluent speakers; LA: lip aperture; TTR: tongue tip retraction;
TT-TD: tongue tip-to-teeth distance; TT-ARD: tongue tip-to-alveolar
ridge distance; TB-PD: tongue body-to-palate distance
