# Supplementary material for: Cluster analysis of articulatory trajectories in fluent nonword productions separates adults who stutter from fluent speakers
Source: Sci Rep. 2025 Nov 4;15:38465. doi: 10.1038/s41598-025-25829-0 (PMC12586618; doi:10.1038/s41598-025-25829-0)
Supplement: Supplementary file 8 — Supplementary Information 8. [file 41598_2025_25829_MOESM8_ESM.pptx]

## Slide 1
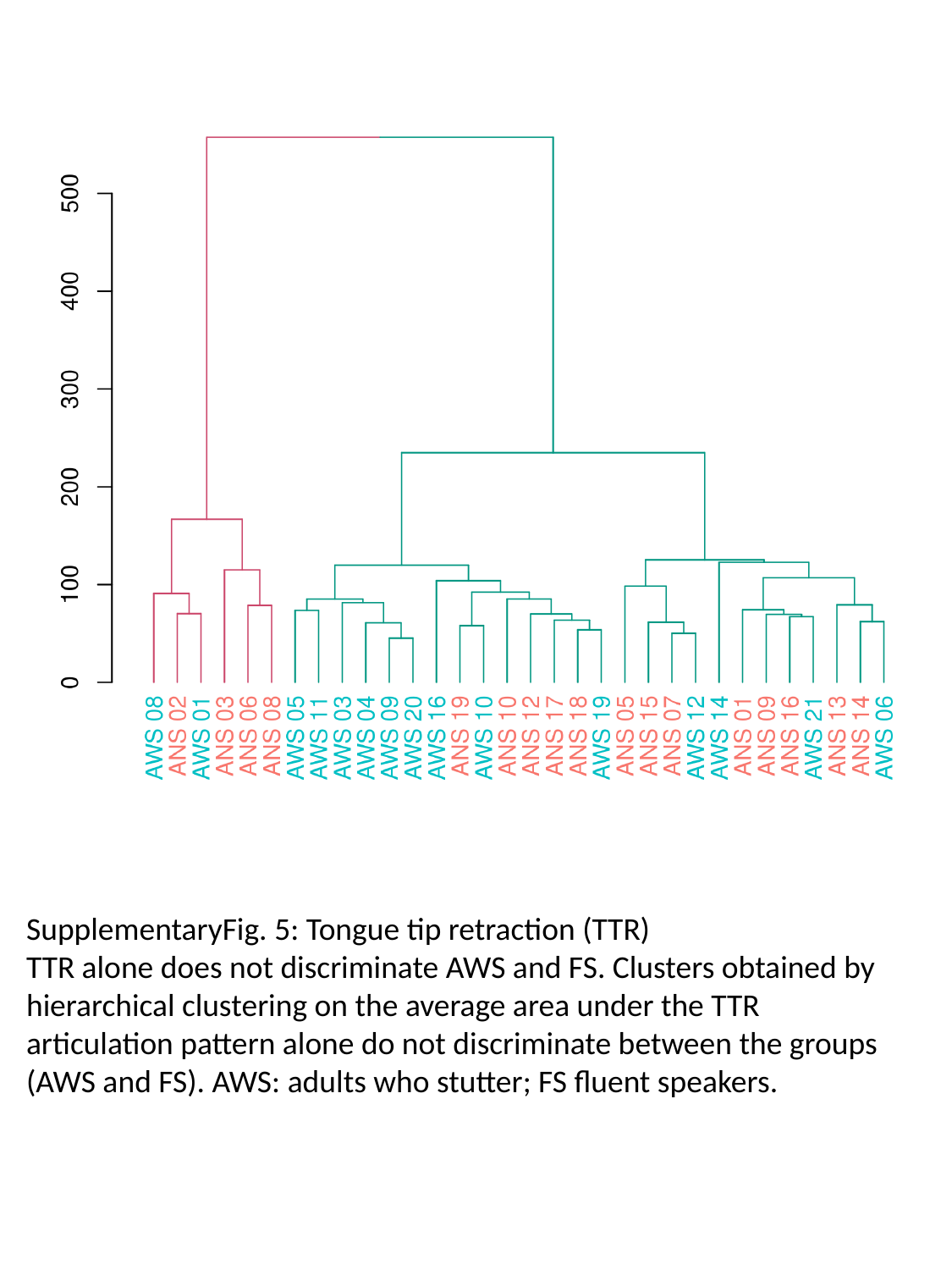

SupplementaryFig. 5: Tongue tip retraction (TTR)
TTR alone does not discriminate AWS and FS. Clusters obtained by
hierarchical clustering on the average area under the TTR
articulation pattern alone do not discriminate between the groups
(AWS and FS). AWS: adults who stutter; FS fluent speakers.
