# Supplementary material for: Cluster analysis of articulatory trajectories in fluent nonword productions separates adults who stutter from fluent speakers
Source: Sci Rep. 2025 Nov 4;15:38465. doi: 10.1038/s41598-025-25829-0 (PMC12586618; doi:10.1038/s41598-025-25829-0)
Supplement: Supplementary file 9 — Supplementary Information 9. [file 41598_2025_25829_MOESM9_ESM.pptx]

## Slide 1
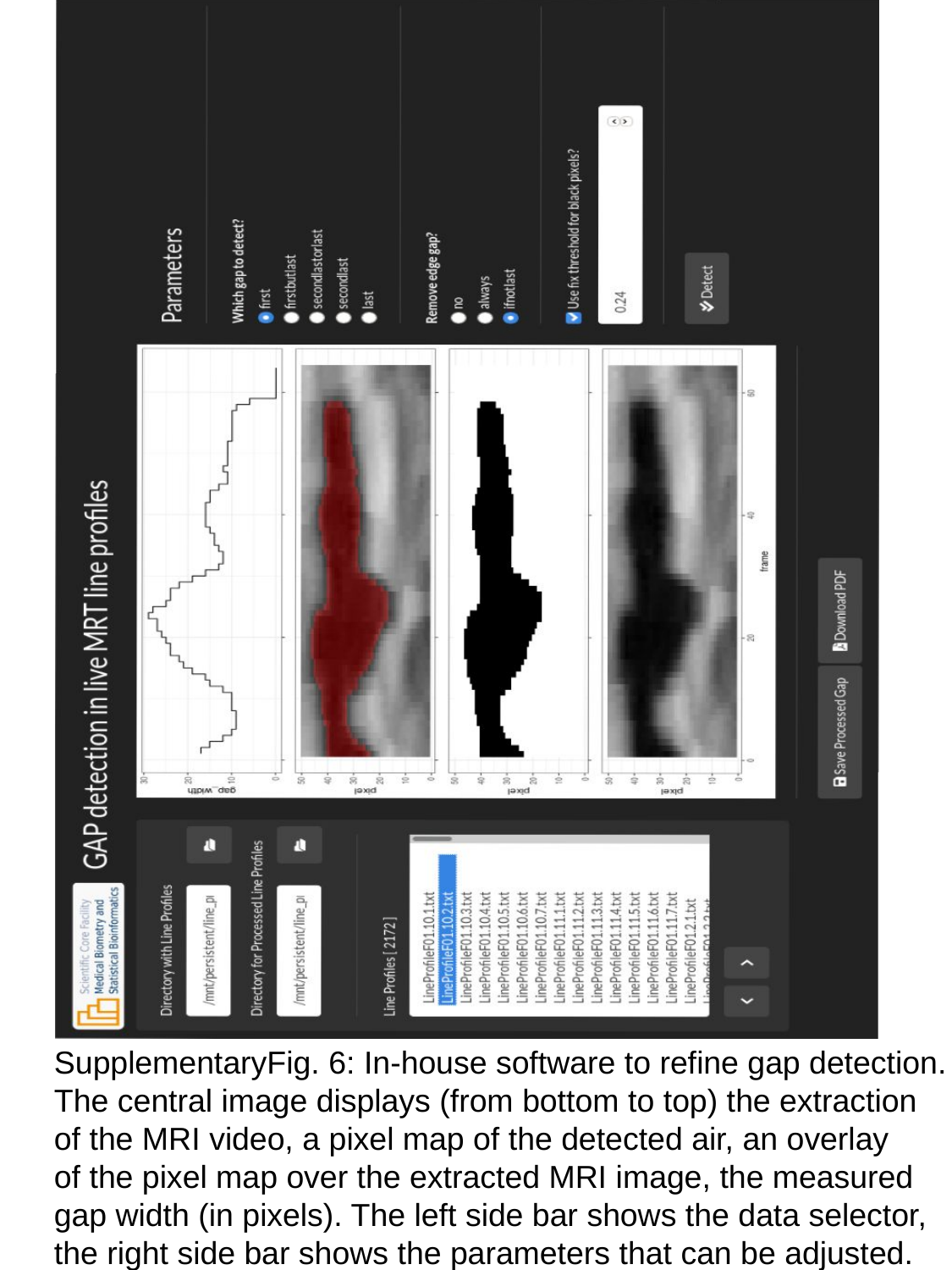

SupplementaryFig. 6: In-house software to refine gap detection.
The central image displays (from bottom to top) the extraction
of the MRI video, a pixel map of the detected air, an overlay
of the pixel map over the extracted MRI image, the measured
gap width (in pixels). The left side bar shows the data selector,
the right side bar shows the parameters that can be adjusted.
