# Supplementary material for: Cluster analysis of articulatory trajectories in fluent nonword productions separates adults who stutter from fluent speakers
Source: Sci Rep. 2025 Nov 4;15:38465. doi: 10.1038/s41598-025-25829-0 (PMC12586618; doi:10.1038/s41598-025-25829-0)
Supplement: Supplementary file 10 — Supplementary Information 10. [file 41598_2025_25829_MOESM10_ESM.pptx]

## Slide 1
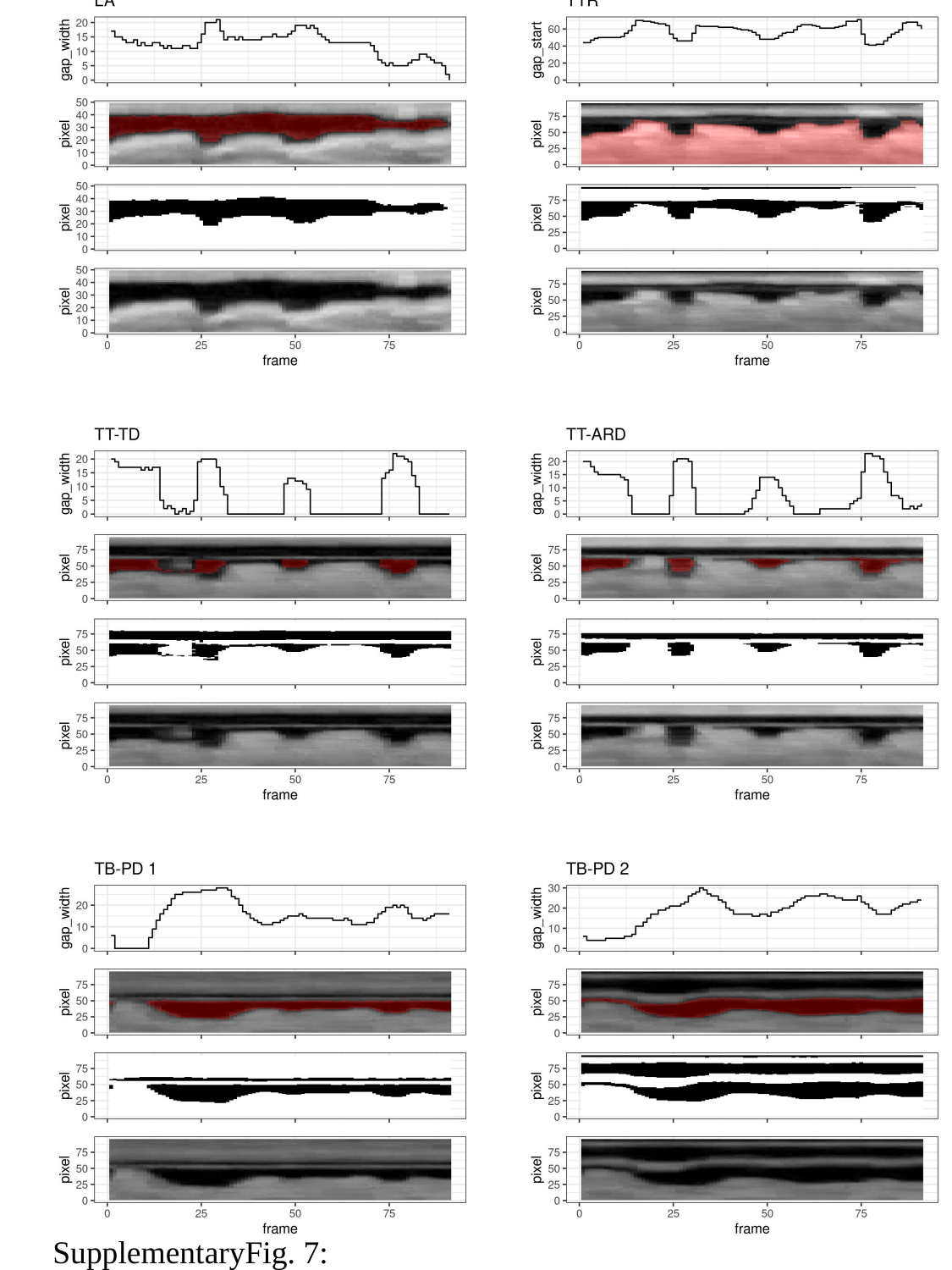

Supplementary Figure 2
SupplementaryFig. 7:

## Slide 2
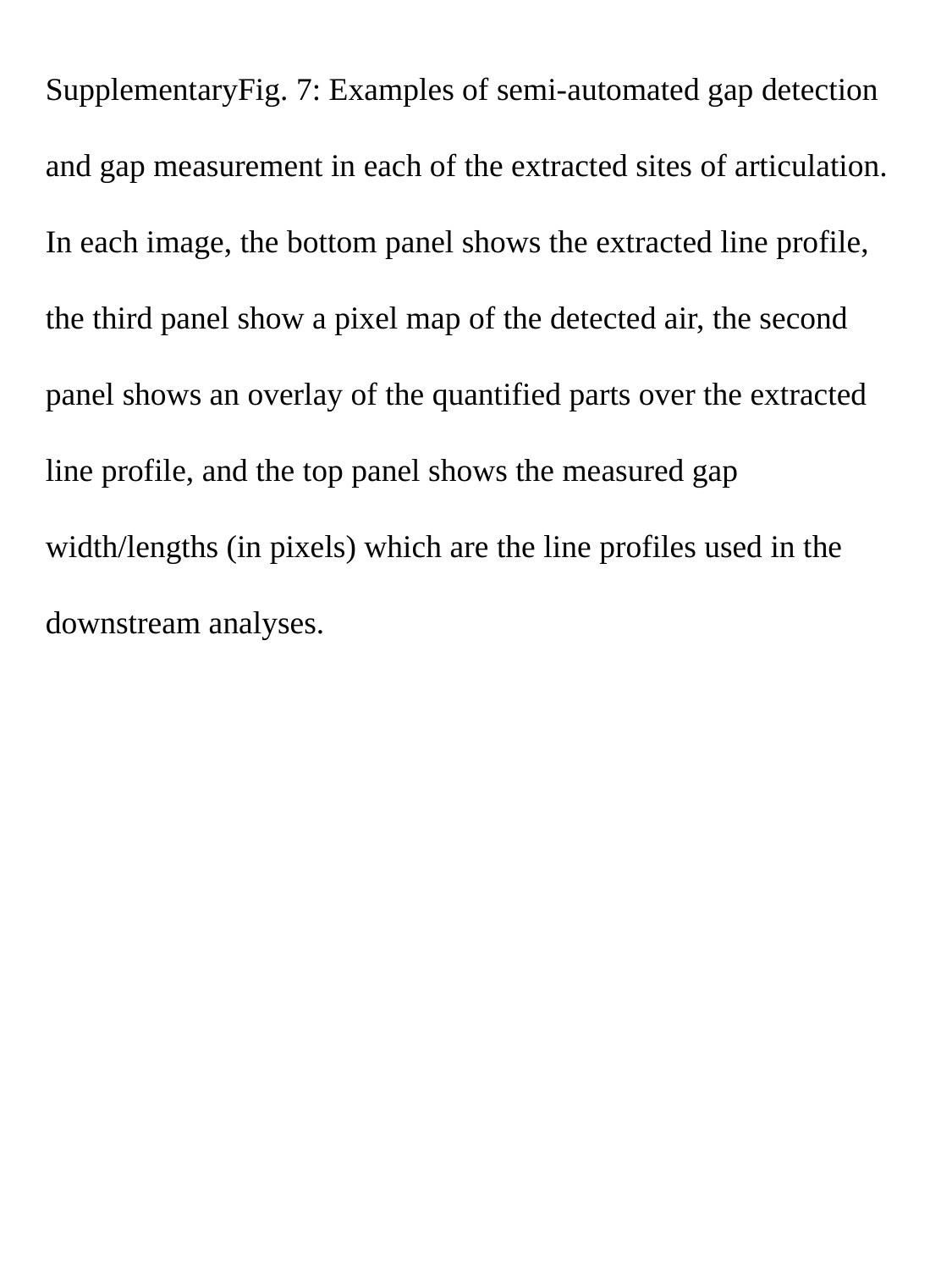

SupplementaryFig. 7: Examples of semi-automated gap detection and gap measurement in each of the extracted sites of articulation. In each image, the bottom panel shows the extracted line profile, the third panel show a pixel map of the detected air, the second panel shows an overlay of the quantified parts over the extracted line profile, and the top panel shows the measured gap width/lengths (in pixels) which are the line profiles used in the downstream analyses.
